# Emotional Eating: A Virtually Untreated Risk Factor for Outcome Following Bariatric Surgery

**DOI:** 10.1100/2012/365961

**Published:** 2012-04-01

**Authors:** Betty E. Chesler

**Affiliations:** Private Practice, 6315 Forbes Avenue, Pittsburgh, PA 15217, USA

## Abstract

Empirical investigations implicate emotional eating (EE) in dysfunctional eating behavior such as uncontrolled overeating and insufficient weight loss following bariatric surgery. They demonstrate that EE may be a conscious or reflexive behavior motivated by multiple negative emotions and/or feelings of distress about loss-of-control eating. EE, however, has not been targeted in pre- or postoperative interventions or examined as an explanatory construct for failed treatment of dysfunctional eating. Three cases suggest that cognitive behavioral treatment (CBT) might alleviate EE. One describes treatment for distress provoked by loss-of-control eating. The first of two others, associated with negative emotions/life situations, link treatment of a super-super-preoperative obese individual's reflexive EE with 52% excess BMI (body mass index) loss maintained for the past year, 64 months after surgery. The second relates treatment of conscious/reflexive EE with 84.52% excess BMI loss 53 months after surgery. Implications for research and treatment are discussed.

## 1. Introduction

Bariatric operations provide the only effective and sustainable means of reducing morbid obesity and related physical maladies [1–3]. Moreover, a growing literature associates bariatric surgery with reduced health care costs [4–7]. Postoperative outcomes, however, vary [8, 9]. Maximum weight loss typically occurs one year after bariatric surgery [10]. Postoperative weight regain emerges 18 to 24 months after surgery [11]. About 20% of postoperative patients experience insufficient weight loss [12]. Insufficient weight loss is frequently defined as less than 50% excess weight loss (<50% EWL) [9, 10, 13].

Weight loss following bariatric surgery is determined by food intake [14]. A French nationwide study of the outcome of all bariatric surgery procedures consecutively performed in a given period found that patients who had not changed their eating habits had a 2.2 times greater risk of failed surgery than those who did [15]. Moreover, many studies link poor weight loss after bariatric surgery to noncompliance with postsurgical dietary recommendations [16–18].

It should be noted that, during the first few years after bariatric surgery, patients typically do not engage in binge eating or consume a large amount of food in a short period of time because of surgery-induced food restriction and the inevitable vomiting that occurs in response to eating too much or too rapidly [19]. Postoperative excessive eating, then, includes such eating patterns as binge eating, defined as loss-of-control eating and eating more than usual [20], graze eating or the repeated consumption of small amounts of food over a long period of time [21], and snack eating or regular consumption of high-fat and sugary foods which fail to meet criterion for binge eating [19].

## 2. EE: A Risk Factor for Negative Postoperative Outcome

Emotional eating (EE), or eating in response to emotional distress, has been found to precede and follow bariatric operations [19, 22] and has been identified by many bariatric surgical programs as a risk factor for poor outcome after bariatric surgery [23, 24]. Clinical interviews with bariatric patients before and after surgery have shown that EE is a crucial factor for outcome following bariatric surgery [25]. One investigation, moreover, demonstrated that EE predicted outcome following bariatric surgery [26].

Reporting that 18% of 500 bariatric candidates failed to be cleared for bariatric operations, one study reported that two of the three most common reasons for that negative recommendation were overeating to cope with emotional distress [24]. The candidates assessed with overeating in response to stress were referred to a psychologist to learn alternate methods of coping with emotional distress. However, neither the specifics nor outcome of that intervention was reported.

## 3. EE Behaviors

EE has been implicated in postoperative graze eating [21, 27–29] uncontrolled overeating [19, 30–32], and snack eating [19, 25].

### 3.1. Conscious and Reflexive EE

In a review of available literature through 2009 that does not address bariatric patients, EE is shown to be a conscious behavior to ease emotional distress, as well as an automatic reaction to unrecognized negative feelings (e.g., reflexive EE). Reflexive EE is reported to be highly associated with alexithymia, a set of cognitive-affective deficits that include difficulties in identifying and communicating feelings, and poor interoceptive awareness, characterized, in part, by difficulty in recognizing and accurately identifying emotions [33].

Some evidence suggests that some bariatric candidates might engage in reflexive EE. A recent study found that morbidly obese females who apply for bariatric surgery reported higher scores on difficulty identifying feelings (alexithymia) and suppression of emotions (interoceptive awareness) than the general population or control group. Moreover, more negative affect and a higher difficulty identifying feelings were correlated with more EE among the morbidly obese women [34].

## 4. EE, Motivation, and Postoperative Weight Loss

EE has been categorized as a means of alleviating a variety of negative moods or distress about losing control of a diet. In one study, pre- and postoperative graze eaters were shown to eat in response to numerous emotional triggers. Uncontrolled eaters and those who engaged in both postoperative uncontrolled eating and grazing, however, ate in response to emotional distress emanating from both multiple negative moods and losing control of eating [27]. In that investigation, uncontrolled eating and grazing after surgery were associated with poorer percent of weight loss (% WL).

Two studies assessing the impact of EE on postoperative binge eating or uncontrolled overeating and weight loss [31, 32] evaluated EE with the Dutch Eating Behavior Questionnaire (DEBQ) (e.g., “Do you have a desire to eat when you are irritated,” “When you are angry do you feel like eating” "Do you want to eat when you are feeling lonely?”) [35]. Both of those studies related postoperative binge eating to a variety of emotional triggers and implicated EE in postoperative weight regain. EE provoked by distress about losing control of eating, however, was not assessed.

The findings from the three studies discussed above [27, 31, 32] suggest that emotional eaters might use food to feel better in reaction to a host of negative feelings and associated situations including distress about loss-of-control eating. Further research, however, might find that some bariatric patients only use food to alleviate distress triggered by their inability to exercise dietary restraint. Therefore, clinical conceptualizations for the treatment of EE might reasonably consider EE motivated by both multiple emotional triggers and distress of losing control over diet.

## 5. EE: A Disconnect between Research and Treatment

The empirical studies discussed above implicate EE in excessive postoperative eating behaviors (graze eating, uncontrolled overeating, and snack eating) and poor postoperative weight loss. They demonstrate that EE may be a conscious or reflexive behavior motivated by multiple negative moods and/or feelings of distress about losing control of a diet. Nevertheless, EE has not been addressed in any pre- or postoperative intervention for the bariatric candidate or patient.

### 5.1. EE: An Unexamined Factor in Failed Treatment

Current interventions for improving postoperative eating and/or weight loss neither discuss or assess for conscious or reflexive EE related to distress about losing control of eating and/or multiple negative feelings/life distress. This section focuses on the possible relevance of those omissions for outcome in two interventions that emphasized strategies for improved eating. Much of the information used in this discussion was derived from a review of postoperative maladaptive eating behaviors observed in the bariatric practice of Rusch and Andris [19].

One study described a brief preoperative cognitive behavioral group intervention for reducing binge eating [36]. In that investigation, 128 women were classified as responders or nonresponders according to postintervention outcomes that measured severity of both subjective binge eating (SBE) behaviors and binge eating symptoms (e.g., loss of control eating and guilt after binge eating). At six and twelve months after surgery, responders to the intervention lost more weight than nonresponders.

In that intervention [36], which reported nearly as many nonresponders (61) as responders (67), the authors speculated about the differences between nonresponders and responders. Those investigators observed that, in contrast to responders, nonresponders appeared to have more severe binge eating attitudes before the intervention. Their study, however, did not consider conscious or reflexive EE, raising the possibility that at least some of the nonresponders' attitudes reflected a historic utilization of binge eating to deliberately or reflexively cope with emotional distress.

Rusch and Andris [19] report that early postoperative treatment for binge eating, emphasizing dietary changes and cognitive behavioral strategies (e.g., hunger control strategies), often offers effective alternatives for bingeing, but some patients eventually resume binge eating (usually on a smaller scale) because life stressors persist and the relief from bingeing continues to impact eating behaviors. Whether or to what extent that resumption of EE is motivated by multiple negative emotions and associated situations or distress about loss-of-control-eating, or both, are unexplored in the literature, but essential to the alleviation of EE.

Another single study illustrates that problem. That investigation analyzed the eating behaviors of a relatively large patient group with both short (8 months through 2 years after bariatric surgery) and long (more than two years after bariatric surgery) follow-up duration [31]. Less binge eating and EE were found in the short term, more binge eating and EE were shown in the long term, but a lower prevalence of binge eating and EE were reported after than that before surgery. EE was measured by the DEBQ [35], which does not assess for distress provoked by out-of-control eating. Because EE increased in the long term when weight gain typically occurs, it is possible that distress about loss-of-control eating might have contributed to the increase in EE and associated binge eating. Bariatric interventions that focus on eating behaviors alone, without treating the distress motivating them, seem to be insufficient for binge eaters who eat to feel better.

One investigation explored the efficacy of a behavioral intervention to decrease caloric intake and increase energy expenditure for postoperative bariatric patients, who had undergone bariatric surgery at or over three years before study entry and had achieved <50% excess weight loss [20]. Study participants were randomly assigned to either a 6-month behavioral intervention or a wait-list control group.

The intervention patients had a greater percentage of excess weight loss than did the wait-list control group at both six months and twelve months after the intervention. However, the differences were not significant. The results, moreover, varied, with some patients continuing to gain weight despite participation in the behavioral intervention. Binge eating, defined as uncontrolled overeating, was assessed, but not related to excess weight loss in either the intervention or the wait-list groups. More importantly, EE was not evaluated, and other eating behaviors associated with EE, such as snacking, were not considered.

Postoperative snack eaters experienced the worst weight loss when compared to normal and sweet eaters at or over one year after bariatric surgery [37]. Rusch and Andris [19] find that a large number of preoperative bariatric patients snack in response to stress/negative emotions. When facing stressful situations postoperatively, they repeat former snacking behaviors within the limits of surgically induced food restriction (e.g., drinking small amounts of rich liquids) and are ultimately shown to resume presurgical patterns of snacking to relieve stress, frustration, and loneliness.

In their bariatric practice, Rusch and Andris [19] assess preoperative candidates for emotional/stress eating that does not meet criterion for an eating disorder. Patients identified with that pattern of eating are evaluated further and/or assigned to a brief intervention before surgery. Unfortunately, the details of that intervention and its impact on postoperative eating were not reported.

Taken together, the studies discussed above suggest that unexamined EE might be one explanatory construct leading to less than optimal results among some participants in some bariatric interventions.

## 6. Treatment of Emotional Eating for the Bariatric Patient

Currently, there are no studies that target conscious or reflexive EE provoked by multiple negative emotional triggers and associated situations and/or distress over losing control of eating in the treatment of dysfunctional eating after bariatric surgery. Three case reports, however, demonstrate how cognitive behavioral therapy (CBT) might address those factors.

The first case, reported by Rusch and Andris [19], described a postoperative intervention of unspecified duration for distress provoked by loss-of-control eating. RS was a 31-year-old single woman, who 36 months after surgery weighed only 30 pounds less than her preoperative weight. Her weight regain was related to a gradual resumption of preoperative snacking in response to feeling depressed, distressed about life issues, and shame about losing control of her diet. In addition to receiving antidepressant medication, that intervention addressed and restructured RS' erroneous beliefs provoking distress about losing control of her diet. Moreover, it fostered patient control over eating by encouraging RS to use stimulus control strategies based on proactive thinking and planned activities such as taking a walk when she normally snacked. That approach engendered increased self-efficacy and a sense of accomplishment, which in turn led to improved mood and an increased desire to pursue weight loss goals. Unfortunately, the eventual impact of that resolve on eating and weight was not reported.

Two case reports, one published [33] and one reported for the first time, were developed in the private practice of this author and are the only accounts of the treatment of EE and associated outcome for the bariatric patient. Weight loss was determined by the formula for percent of excess BMI lost (%EBL): %EBL = [(preoperative  BMI − current  BMI)/(preoperative  BMI − 25)] × 100, as suggested by [38].

The published case report describes comprehensive long-term pre- and postoperative treatment for emotional eating and related postoperative weight loss for a Caucasian, working married mother, 56 years old, BMI of 44.97 at intake. Therapy focused on conscious and sometimes reflexive EE provoked by multiple negative emotional triggers and related life situations. It was associated with 84.52% excess BMI (body mass index) loss 53 months after surgery.

That case focused on emotional eating beliefs (EEB, that is, equations of food with both alleviation of negative feelings and friendship) and reactance to dietary advice (RDA, that is, rebelling against prescribed nutrition) for the deliberate use of food to feel both better and befriended. EEB and RDA were shown to motivate and justify, respectively, the conscious consumption of rich or “forbidden foods.” Therapy explored and repudiated the irrational assumptions of EEB. It emphasized coping with emotional distress emanating from life issues through rational thinking and problem solving and spousal/social support, instead of eating. RDA treatment emphasized awareness of healthy preoperative and appropriate postoperative eating and encouraged the patient to develop and discuss satisfying ways of fulfilling dietary prescriptions. Adherence to prescribed antidepressants, eating behaviors, and weight were monitored at each therapy session.

The unpublished case suggests that postoperative treatment focused on detecting and coping with emotional distress and reactance to dietary advice may reduce reflexive EE and stabilize weight loss among preoperative super-super-obese (BMI = ≥60 kg/m^2^) individuals. At intake, O was a 33-year-old Caucasian postoperative married father and business man, BMI of 33.57. He reported undergoing bariatric surgery three years ago when his BMI was 65.05.

Despite O's significant weight loss, he obsessed about overeating and regaining presurgical weight. Eating sparingly, with occasional bouts of overeating during the day, O reported eating a small dinner at home alone because he feared that dining with his family would cause him to overeat. He also indicated taking pain pills which proved to be a coping mechanism for unrecognized emotional distress.

O has been in treatment for two years and four months. He stopped taking pain pills about two months after the onset of therapy. Over time, he has learned to identify and cope with emotional distress provoked by life stress through rational thinking and problem solving. O feels easier about life situations that used to bother him in association with a stronger and closer relationship with his wife. O controls his reactance to dietary advice, for example, by telling himself, “I do not have to eat both things. I can eat one today, and the other tomorrow.” O regained weight during the first year and four months of therapy. For the past year, however, O has stabilized and maintained a BMI of 44.25. He experienced a 52% excess BMI loss, 64 four months after surgery.

Those results, indicative of successful weight loss for all bariatric patients, may be more significant for preoperative super-super-obese patients who are shown to achieve less excess weight loss than patients with lower preoperative BMIs [15, 39, 40]. Similarly, male gender is shown to be a predictive factor for poor weight loss after gastric banding [41].

O's outcome suggests that comprehensive treatment might successfully be administered after bariatric surgery. In support of that idea, one study finds that when compared to preoperative candidates, postoperative patients are more likely to start, attend, and complete a behavioral intervention [42].

## 7. Conclusion: Implications for Research and Treatment

EE can be reflexive or conscious, emanate from multiple negative emotions or distress over loss-of-control eating, trigger dysfunctional eating, and affect less than optimal weight loss after bariatric surgery. The response to and utilization of that information, however, is associated with only two published case reports and one unpublished case. One of those cases discusses treatment for distress triggered by loss-of-control eating after bariatric surgery. Two cases associate successful postoperative weight loss with the treatment of reflexive EE in one case and conscious/reflexive EE in the other, provoked in both instances by emotional distress rooted in a variety of life stressors.

The information reported in this paper suggests that untreated EE is a risk factor for poor postoperative weight loss and associated medical maladies and health care costs. Surgery combined with treatment for EE might provide the only opportunity for bariatric candidates who engage in EE to optimize outcomes. Central to treating EE are assessments for conscious and reflexive EE, evaluations for EE triggered by multiple life stressors and loss-of-control eating, and the development of varied coping strategies for emotional distress.

The greatest innovations in psychotherapeutics have emerged from the case report (e.g., Aaron Beck) [43]. The three reported cases could be the basis for empirical research whose findings might recommend adjustments and/or the incorporation of additional strategies to accommodate to specific EE profiles such as reflexive EE associated with a particular set of life stressors.
